# Effects of Excess Energy Intake on Glucose and Lipid Metabolism in C57BL/6 Mice

**DOI:** 10.1371/journal.pone.0146675

**Published:** 2016-01-08

**Authors:** Jing Pang, Chao Xi, Xiuqing Huang, Ju Cui, Huan Gong, Tiemei Zhang

**Affiliations:** 1 The Key Laboratory of Geriatrics, Beijing Hospital & Beijing Institute of Geriatrics, Ministry of Health, Beijing, China; 2 College of Life Sciences, Beijing Normal University, Beijing, China; The Ohio State University, UNITED STATES

## Abstract

Excess energy intake correlates with the development of metabolic disorders. However, different energy-dense foods have different effects on metabolism. To compare the effects of a high-fat diet, a high-fructose diet and a combination high-fat/high-fructose diet on glucose and lipid metabolism, male C57BL/6 mice were fed with one of four different diets for 3 months: standard chow; standard diet and access to fructose water; a high fat diet; and a high fat diet with fructose water. After 3 months of feeding, the high-fat and the combined high-fat/high-fructose groups showed significantly increased body weights, accompanied by hyperglycemia and insulin resistance; however, the high-fructose group was not different from the control group. All three energy-dense groups showed significantly higher visceral fat weights, total cholesterol concentrations, and low-density lipoprotein cholesterol concentrations compared with the control group. Assays of basal metabolism showed that the respiratory quotient of the high-fat, the high-fructose, and the high-fat/high-fructose groups decreased compared with the control group. The present study confirmed the deleterious effect of high energy diets on body weight and metabolism, but suggested that the energy efficiency of the high-fructose diet was much lower than that of the high-fat diet. In addition, fructose supplementation did not worsen the detrimental effects of high-fat feeding alone on metabolism in C57BL/6 mice.

## Introduction

Obesity is an independent high risk factor for metabolic diseases, such as type 2 diabetes and nonalcoholic fatty liver disease. The constituents of our modern diet have changed; therefore, the prevalence of nutritional imbalance-induced obesity is increasing [1]. Excess energy intake is thought to be a major contributor to obesity and metabolic disorders.

Mice fed with high energy food are used as a model system to understand the mechanisms of the impairment of metabolic homeostasis. *Ad libitum* access to a high-fat-diet in mice induced insulin resistance, hyperglycemia and dyslipidemia [2]. In addition to high fat food, fructose, which exists in many fruits, honey and high fructose corn syrup, is consumed in large amounts by humans [3]. Increasing evidence suggests that high consumption of fructose can also lead to insulin resistance and liver steatosis [4, 5].

In addition to the composition of high energy foods, different quantities of fat and fructose were added to the diet to investigate the detrimental effects of overeating energy-dense foods. Mice fed with either 60% fructose-enriched diet or 20% fructose in drinking water developed glucose intolerance and dyslipidemia [6, 7]. A high-fat-diet (containing 60% fat) is usually used to produce animal obesity models to observe the consequences of nutrient imbalance [8]. Recently, many studies have combined a high fat diet and high fructose water to induce the main features of human metabolic disorders [9, 10].

According to these studies, different quantities and compositions of energy-dense foods can increase weight gain and insulin resistance. However, whether increased calorie intake induces more detrimental effects, and which kinds of energy supplier are more efficient to induce metabolic dysfunction still need to be investigated. To compare the effects of different energy compositions and quantities on metabolic homeostasis, we examined the effects of a high-fat diet, a high-fructose diet and a combination high-fat/high-fructose diet on glucose and lipid metabolism in C57BL/6 mice. This study allowed us to evaluate the extent of metabolic disturbance under different energy states induced by different materials, and provides clues to choose a better animal model for future research.

## Materials and Methods

This study was carried out in strict accordance with the recommendations in the Guide for the Care and Use of Laboratory Animals of the National Institutes of Health. The protocol was approved by the Biomedical Ethics Committee of Beijing normal University. All surgery was performed under 10% chloral hydrate anesthesia, and all efforts were made to minimize animal suffering.

### Animals and diets

Six—eight week-old, male mice (C57BL/6J strain) were housed in plastic cages containing wood shavings and maintained in a room with a 12h-light cycle at room temperature 22 ± 2°C and a humidity of 50 ± 5%, with free access to food and tap water. Mice were adapted to these conditions for 1 week. During adaptation the mice were healthy and none died or were injured.

Mice were separated into four groups of nine mice each: (1) normal fat diet (3.42 kcal/g; GB14924, Huafukang, China) with normal drinking water (control); (2) normal fat diet with 20% D-fructose (0.8 kcal/ml; Amresco, USA) drinking water (HFR); (3) high fat diet (5.24 kcal/g; D12492, Huafukang) with normal drinking water (HFA); and (4) high fat diet with 20% fructose drinking water (HFF). The compositions of the experimental diets are shown in Table 1. All mice received food and water *ad libitum* for 3 months. Body weight was monitored at 1 week intervals throughout the whole study. Food and water consumption were measured every two weeks. The energy efficiency of body weight was calculated as weight gained (mg) divided by energy intake (kcal) in the period from 0 to 4 weeks, 4 to 8 weeks and 8 to 12 weeks, respectively.

**Table 1:** Composition of experimental diets.

Component	Normal diet	High-fat diet
Carbohydrate (%)	65.42	20
Protein (%)	22.47	20
Fat (%)	12.11	60
Vitamins	+	+

### Intraperitoneal glucose tolerance test (IpGTT)

The animals were fasted for 12 hours. After estimating basal glucose levels, the animals received an intraperitoneal injection of 2 g glucose/kg body weight. Blood samples were collected from the tip of the tail at intervals of 15, 30, 60 and 120 min. The blood glucose was measured using a Bayer, Brio blood glucose meter. The area under the curve was calculated as an index for whole-body insulin sensitivity. Insulin sensitivity was also evaluated by the glucose disappearance rate, which was expressed as glucose fall per minute (mmol/L/min). In the IpGTT experiment of control mice, the blood glucose concentration began to fall at 15 min post-glucose injection. Thus, the glucose disappearance rate was calculated by glucose concentration fall divided by time, in the periods from 15 to 30 min, 30 to 60 min, and 60 to 120 min post-glucose injection.

### Intraperitoneal insulin tolerance test (IpITT)

Baseline blood glucose was measured following a 4 hour fast from 9.00 a.m. to 1.00 p.m. Mice were then given an intraperitoneal injection of 0.5 U insulin/kg body weight. Glucose was then measured in tail-tip blood samples at intervals of 15, 30, 60 and 120 min. The area under the curve below baseline glucose was calculated as an index for whole-body insulin sensitivity. During each period post-insulin injection (0–15min, 15–30min, 30–60min, 60–120min), the glucose disappearance rate was also calculated as a measure of insulin sensitivity.

### Blood samples and tissue collection

After 3 months of treatment, mice were fasted overnight, after which blood glucose was monitored using a glucometer (Bayer). The whole blood sample was collected by retro-orbital bleeding under chloral hydrate anesthesia. Serum was isolated by centrifugation and aliquots were stored at −80°C before analysis. Triglycerides, total cholesterol, high-density lipoprotein cholesterol, low-density lipoprotein cholesterol, aspartate aminotransferase (AST) and alanine aminotransferase (ALT) were measured by an automatic analyzer (Hitachi, 7600, 7170A). The liver and visceral fats were removed, weighed and stored in liquid nitrogen.

### Insulin and insulin sensitivity indices

Serum insulin was assayed using a commercial enzyme linked immunosorbent assay (ELISA) kit (Alpco, USA). Insulin resistance was assessed by homeostatic model assessment-insulin resistance (HOMA-IR), quantitative insulin sensitivity check index (QUICKI) and the fasting insulin resistance index (FIRI) [11]. The formulae are given below:

HOMA-IR =  (FBI (μU/ml) × FBG (mmol/L))/22.5

QUICKI = 1/(Log(FBG mg/dL)+ log(FBI μU/ml))

FIRI = (FBI (μU/ml) × FBG (mg/dL))/25

FBI: fasting blood insulin; FBG: fasting blood glucose.

### Oil Red O staining

Liver tissues were fixed in 4% paraformaldehyde and sliced with a microtome to 10 μm in thickness. Liver slides were stained with 0.5% oil red O (Sigma-Aldrich) in isopropanol for 15 min, rinsed in distilled water, sealed with glycerol and examined under a light microscope.

### Metabolic measurements

Mice were individually housed in the metabolic cages (Oxylet), and acclimatized for 24 hours before recording. Their 24-hour oxygen consumption (VO_2_), carbon dioxide production (VCO_2_), respiratory quotient (RQ) and energy expenditure (EE) were measured every hour for 3 min in each cage. Mice were maintained on their energy-dense diet or water throughout the detection process. The metabolic rate for each mouse was calculated as an average of the measurements during the day (7:00–17:00) or night (18:00–6:00).

### Statistical analysis

Data were expressed as mean ± standard deviation (SD). For multiple comparisons among different groups of data, significant differences were determined by one-way analysis of variance (ANOVA) with Bonferroni post-hoc analysis using SPSS version 17.0. To test for differences across time, a one way repeated measures ANOVA with post-hoc tests was used. The difference between the values was considered significant when P < 0.05.

## Results

### Effect of diet on body weight, food intake and energy intake

**Fig 1:**
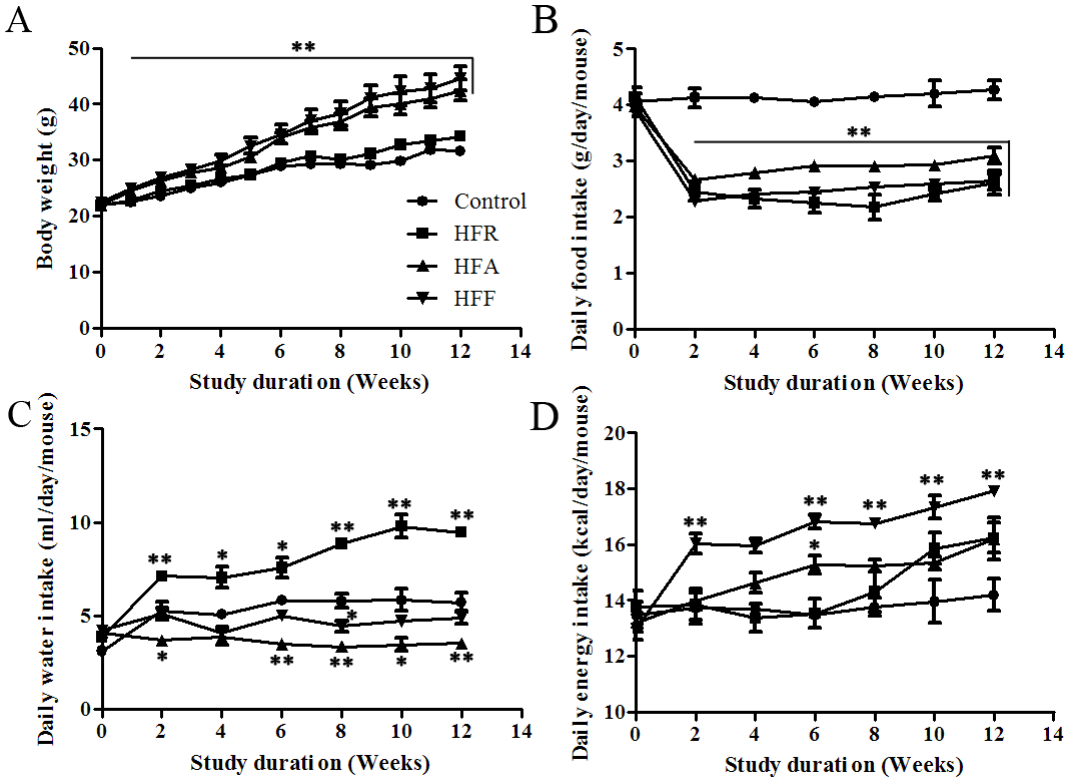
Effect of diet on body weight and energy intake. The C57BL/6 mice were fed with 20% fructose water (HFR), 60% fat diet (HFA), and 20% fructose water + 60% high fat diet (HFF) for 3 months. Body weights (A) were measured every week; food intake (B), water intake (C), and energy intake (D) were measured every other week. Data are presented as means ± SD (n ≥ 6 in each group). The following abbreviations are used throughout the figures: control, mice fed with normal water and diet; HFR, mice fed with 20% fructose water; HFA, mice fed with 60% fat diet; HFF, mice fed with 20% fructose water and 60% fat diet. *P<0.05 versus control at the same time point, **P<0.01 versus control at the same time point.

Compared with the mice in the normal fat group, body weights of the mice in the HFA and HFF groups increased by 1.3-fold and 1.4-fold after 3 months of diet modification (P < 0.05, Fig 1A). There was no significant difference in body weight between the normal fat group and the high fructose group or between the high fat group and high fat-high fructose group throughout the entire 3-month period. After 3 months of feeding, food intake in grams per mouse per day was similar for the HFR, HFA and HFF groups, and all of them were less than the control group (P < 0.05, Fig 1B). Water intake in milliliters per mouse per day by the high fructose group was much higher than in the other groups (P < 0.05, Fig 1C). In addition, there was an increasing trend in the amount of water intake from week 8 in the high fructose feeding mice.

Both the high fat feeding and high fructose feeding groups increased their total calorific intake. Compared with the normal diet group, daily energy intakes at 3 months were 14%, 14% and 26% higher in the HFR, HFA and HFF groups, respectively (P < 0.05, Fig 1D). However, the HFR mice showed higher calorific intake than the control only from week 10, because they increased their intake of fructose water and food from week 8.

### Effect of diet on energy efficiency

The high fructose group showed a significant increase in energy intake from week 8; therefore, we separated the whole experimental period into three phases (0–4 weeks, 4–8 weeks and 8–12 weeks). Energy efficiency was calculated by the gain of body weight from taking 1 kcal from food during these three phases. Mice fed with either the high fat or high fat-high fructose diet for 3 months showed higher energy efficiency than those fed with the normal diet. Compared with that at 0–4 weeks, energy efficiency during 8 to 12 weeks decreased by 46%, 23%, 26% and 28% in the control, HFR, HFA and HFF groups, respectively. At 12 weeks, the HFR, HFA and HFF groups all showed higher energy efficiency than the control group (Table 2). There was no difference in energy efficiency between the HFA and HFF groups.

**Table 2:** Energy efficiency at different time points in each group.

Study duration	0–4 w(mg/kcal)	4–8 w(mg/kcal)	8–12 w(mg/kcal)
Control	10.4 ± 0.6	8.7 ± 1.5	5.7 ± 1.5
HFR	12.2 ± 1.2	9.2 ± 2.3	9.5 ±3.5
HFA	17.2 ± 0.6**	19.3 ± 3.6 **	12.8 ± 2.0*
HFF	17.8 ± 3.2**	18.0 ± 2.0*	12.8 ± 0.5*

Note: Data were presented as means ± SD (n ≥ 6 each group).

*P<0.05 versus control;

**P<0.01 versus control.

### Effect of diet on lipid metabolism and liver function

**Fig 2:**
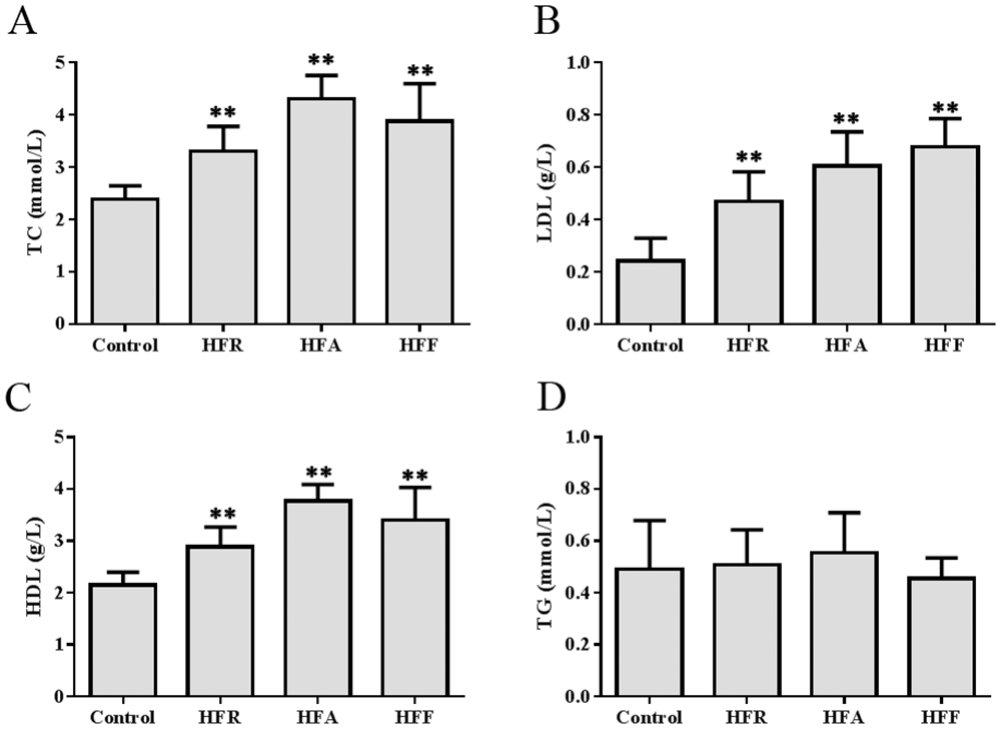
Effect of diet modification on lipid metabolism. The levels of total cholesterol (TC, A), low-density lipoprotein cholesterol (LDL, B), high-density lipoprotein cholesterol (HDL, C) and triglyceride (TG, D) were detected in the control, HFR, HFA and HFF groups. Data are presented as means ± SD (n ≥ 6 in each group). A significant difference was determined by one-way analysis of variance. *P < 0.05 versus control, **P < 0.01 versus control.

**Fig 3:**
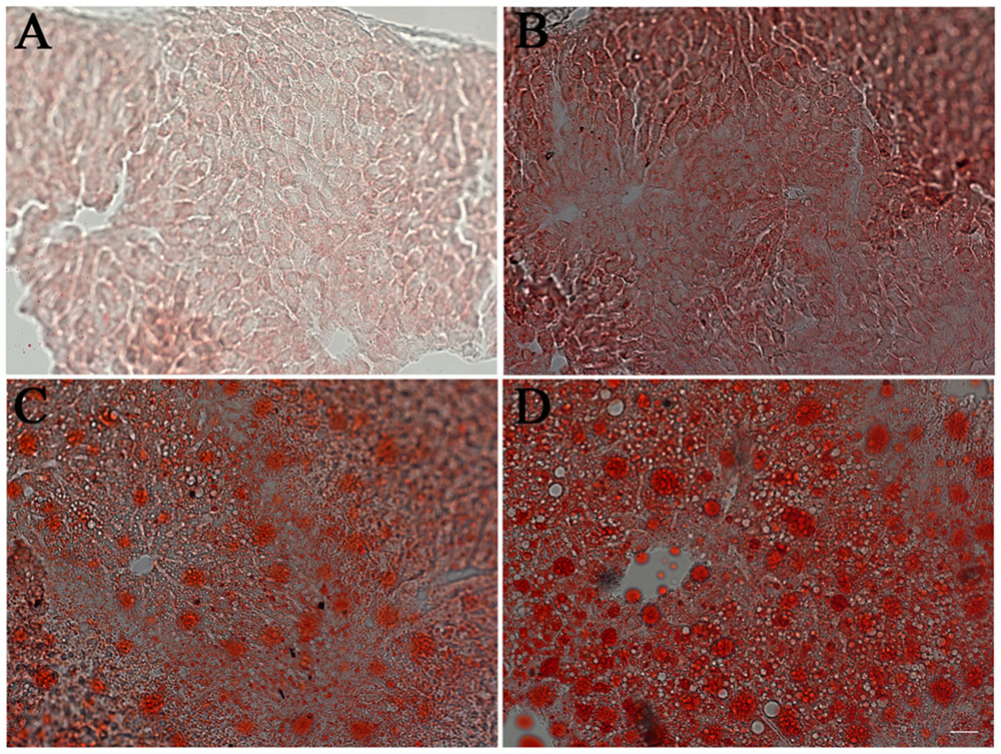
Effect of diet modification on lipid accumulation in the liver. Liver tissue slides were stained with oil red O and photographed under a microscope. Compared with control mice (A), lipid droplets could be detected obviously in the HFR mice (B), HFA mice (C) and HFF mice (D). The length of the scale is 20 μm.

Compared with the serum lipid profiles in the control mice, the total cholesterol levels were elevated by 40% in HFR mice, 80% in HFA mice and 60% in HFF mice (P < 0.05, Fig 2A). Low-density lipoprotein cholesterol increased in HFR mice (by 1.9-fold), HFA mice (by 2.5-fold) and HFF mice (by 2.8-fold) (P < 0.05, Fig 2B). High-density lipoprotein cholesterol also increased in the HFR, HFA and HFF mice (Fig 2C). However, there was no significant difference in triglyceride levels among those groups (Fig 2D). We also examined lipid accumulation in the liver. As shown in Fig 3, compared with the control group, the HFR, HFA and HFF groups all showed much more lipid droplet distribution in their liver tissues. The lipid droplets in the livers of the HFA and HFF mice were much larger than those of the HFR mice. Meanwhile, the amount of visceral adipose (epididymal and perirenal fat weight) also significantly increased in the HFR, HFA and HFF mice (P < 0.05, Table 3). Differences in the wet weight of the liver were not statistically significant among those groups (Table 3). However, the serum AST levels increased by approximately 10% in the HFR group, 40% in the HFA group (P < 0.05) and 60% in the HFF group (P < 0.05) compared with the control group (Table 3).

**Table 3:** Change of visceral fat weight, liver weight and function by diet modifications.

	Control	HFR	HFA	HFF
Epididymal fat weight (g)	0.57±0.15	0.92±0.36**	1.9±0.26**	1.91±0.26**
Perirenal fat weight (g)	0.19±0.05	0.43±0.19	1.05±0.17**	1±0.35**
Liver weight (g)	1.14±0.1	1.3±0.11	1.33±0.09	1.36±0.45
ALT (U/L)	24.73±4.7	19.34±4.2	31.59±10.8	26.68±5.5
AST (U/L)	69.41±6.4	76.16±13.3	96.14±15.1**	109.47±23.4**

Note: Data were presented as means ± SD (n ≥ 6 each group).

**P<0.01 versus control.

### Effect of diet on insulin sensitivity

Among the four groups, the fasting blood glucose level was highest in high fat mice (5.7 ± 1.2 mM) and lowest in the high fructose mice (3.6 ± 0.4 mM, Table 4). The serum insulin levels increased by 1.1-, 2.3- and 1.4-fold in the HFR, HFA (P < 0.05) and HFF groups, respectively, compared with the control group (Table 4). The classic indexes (HOMA, QUICKI, FIRI) used to evaluate insulin sensitivity were calculated using the fasting blood glucose concentration and the fasting serum insulin concentration, and only the HFA group showed marked insulin resistance compared with the control group (P<0.05, Table 4).

**Table 4:** Change of serum insulin and glucose concentrations by diet modifications.

	Control	HFR	HFA	HFF
Insulin (pmol/L)	42.3±17.3	46.7±25	95±47.2*	60.7±25.7
Glucose (mmol/L)	4.3±0.6	3.6±0.4	5.7±1.2*	4.8±1.0
HOMA-IR	1.1±0.46	1.0±0.4	3.5±1.6**	1.9±1.1
FIRI	18.6±7.4	16.6±6.5	56.5±26.7**	31.3±17.5
QUICKI	0.38±0.02	0.39±0.02	0.32±0.02**	0.35±0.03

Note: Data were presented as means ± SD (n ≥ 6 each group).

*P<0.05 versus control.

**P<0.01 versus control.

**Fig 4:**
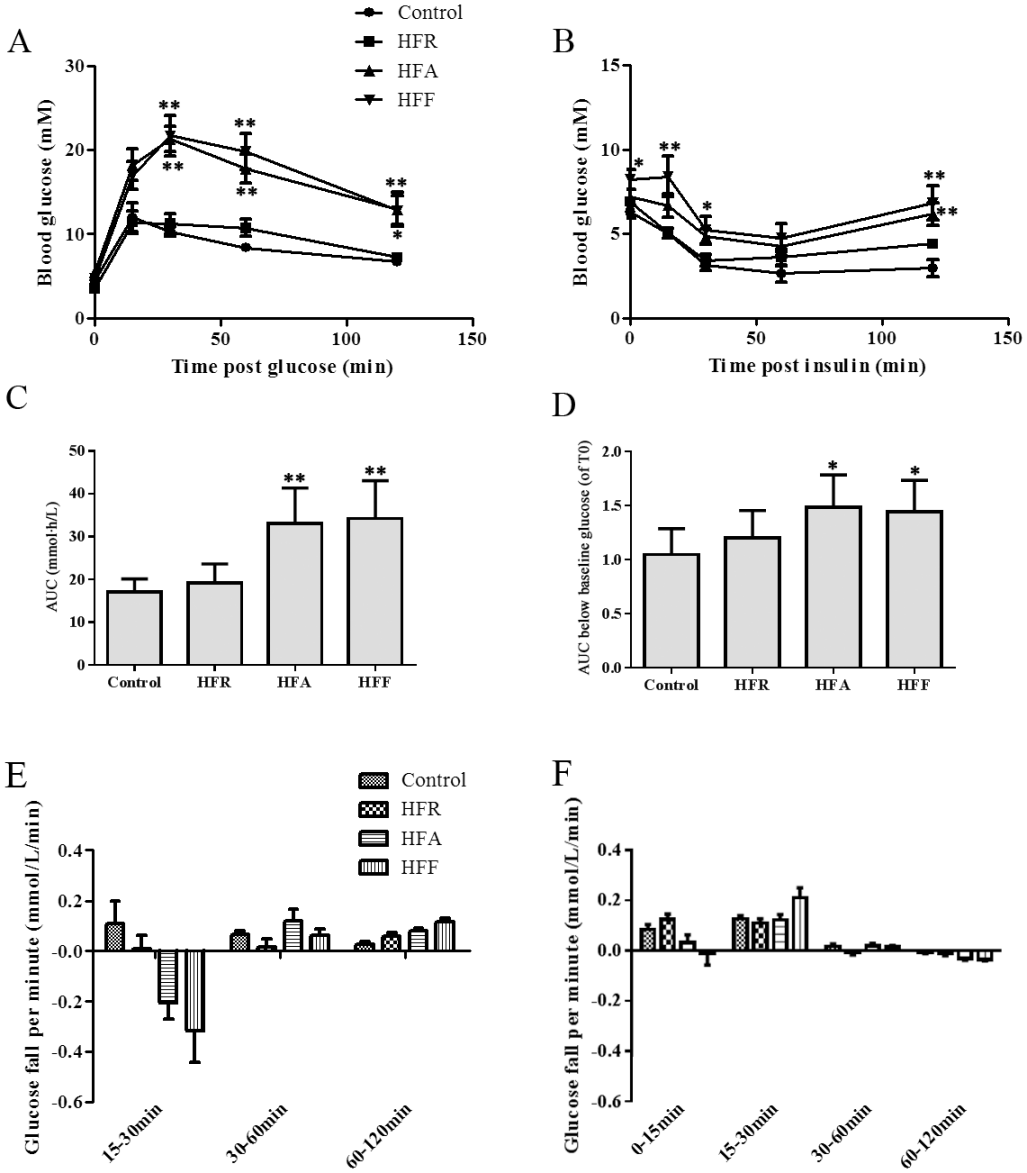
Effect of diet modification on insulin sensitivity. Mice fed with fructose water or high fat diet were subjected to intraperitoneal glucose tolerance tests (A) and intraperitoneal insulin tolerance tests (B). The areas under the curve (AUC, C and D) and the glucose disappearance rate (E and F) were calculated to detect differences among the groups. Data are presented as means ± SD (n ≥ 6 in each group). A significant difference was determined by one-way analysis of variance. *P < 0.05 versus control at the same time point, **P < 0.01 versus control at the same time point.

Inconsistent with the results of the insulin sensitivity indexes, the glucose tolerance and insulin tolerance in the HFA and HFF group were severely impaired (P < 0.05, Fig 4A and 4B). Compared with the normal group (17.1 ± 3.1 mmol·h/L), the area under the curve of plasma glucose following an intraperitoneal injection of glucose was markedly increased in the HFA (33 ± 8.4mmol·h/L) and HFF (34.2 ± 8.8 mmol·h/L) groups (Fig 4C). In the IpITT experiment, the baseline glycemia after fasting for 4 hours was markedly higher in the HFR mice (6.9 ± 0.8 mmol/L), HFA mice (7.2 ± 1.4 mmol/L) and HFF mice (8.2 ± 1.5 mmol/L) compared with the control mice (6.3 ± 1.2 mmol/L). For comparison of glucose response over time relative to the baseline glucose levels, we divided the plasma glucose at each time point by the initial fasting glucose level. And the inverse area under the curve below baseline glucose was significantly larger in the HFA and HFF groups than in the control (P<0.05, Fig 4D).

The glucose disappearance rates at different time points post-glucose or insulin injection were detected, which provided more details about the effect of insulin in the mice. In the IpGTT experiment, the blood glucose concentration of the control mice started to decrease from 15 min and the glucose disappearance rate was fastest in the 15–30 min phase (0.11 mmol/L/min, Fig 4E). However, the glucose concentration did not fall in HFR, HFA and HFF groups during the 15 to 30 min phase (Fig 4E). The highest glucose disappearance level of the HFR and HFF mice occurred during the 60–120min phase (0.06 mmol/L/min and 0.12 mmol/L/min, respectively). Compared with the control group, the three energy-dense groups all showed glucose disappearance delay. In the IpITT experiment, the glucose disappearance rate during the 0–15 min period was higher in the control mice (0.08 mmol/L/min) and HFR mice (0.12 mmol/L/min) than in the HFA mice (0.03 mmol/L/min) and HFF mice (-0.01 mmol/L/min) (Fig 4F). The insulin action during the first 15min post-insulin injection in the HFF group was much lower than that in other three groups, showing a significant delay in glucose disappearance.

### Effect of diet on the RQ and EE values

**Fig 5:**
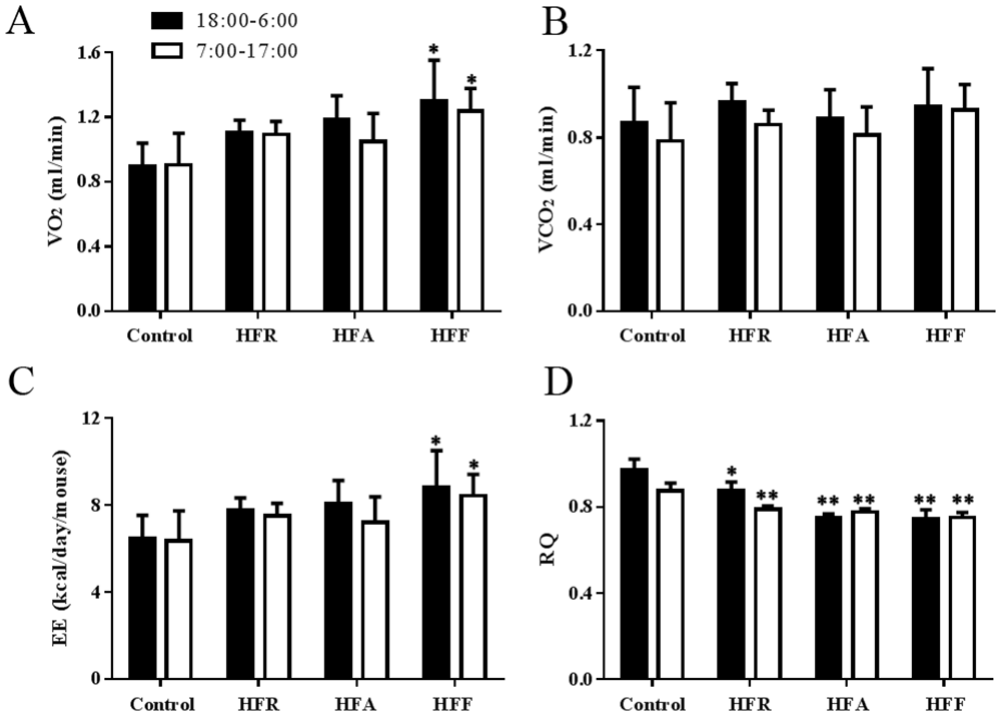
Effect of diet modification on the basal metabolic rate. The levels of the consumed O_2_ (VO_2_, A), the produced CO_2_ (VCO_2_, B), energy expenditure (EE, C), and respiratory quotient (RQ, D) were examined in the control, HFR, HFA and HFF mice. Data are presented as means ± SD (n = 5 in each group). A significant difference was determined by one-way analysis of variance. *P<0.05 versus control in the same phase, **P<0.01 versus control in the same phase.

The RQ is commonly used to evaluate the change in the substrate utilization of energy metabolism; therefore, we examined the levels of VO_2_, VCO_2_, RQ and EE in the groups (Fig 5). Mice fed with the high fat-high fructose diet for 3 months underwent a significant increase in VO_2_ (1.3 ± 0.25 ml/min in the night and 1.24 ± 0.14 ml/min in the day) compared with the control (0.89 ± 0.15 ml/min in the night and 0.9 ± 0.2 ml/min in the day). There was no significant difference in VCO_2_ among these groups. Accordingly, the RQ (expressed as VCO_2_/VO_2_) during the night was significantly decreased, by 10%, in the HFR mice, by 23% in the HFA mice and by 24% in the HFF mice compared to that in the control mice. Higher energy expenditure was also observed in the HFR mice (7.6 ± 0.4 kcal/day), HFA mice (7.7 ± 1.1 kcal/day) and HFF mice (8.7 ± 1.3 kcal/day) compared with the control mice (6.4 ± 1.2 kcal/day).

## Discussion

Excess energy intake is related to the development of metabolic disorders. In this study, 20% fructose water and 60% fat diet as energy sources were supplied to C57BL/6 mice, and the effects of diet modification on glucose and lipid metabolism were compared. We found that (1) the high fat-high fructose diet did not induce more deleterious effects on glucose and lipid metabolism than the high fat diet only, even though the mice consumed more calories. (2) Saturated fat has stronger effects than fructose on the induction of metabolic dysfunction, even though they supplied similar numbers of calories.

Diets high in calories are associated with obesity. However, the present results showed that *ad libitum* access to 20% fructose through drinking water in C57BL/6 mice did not increase weight gain, which was also observed in previous studies [12, 13]; however, this result was inconsistent with some studies performed in rats or hamsters [4, 14]. Not discounting the strain and species differences in susceptibility to the effect of fructose, we revealed that fructose administrated through drinking water limited the amount of energy ingested. After eight weeks of fructose water feeding, the amount of energy taken in did not increase in the fructose-fed mice compared with the control mice. Therefore, a longer period of fructose feeding might have a stronger effect on body weight.

The “Energy efficiency of body weight” calculation has been used previously [15, 16] and provides a convenient way to compare the effects of different energy intake patterns. A previous study [15] reported that energy efficiency increased gradually during feeding with a high fat diet within the first 7 weeks and then began to decrease, which was consistent with our results. In addition, we found that the energy efficiency of fructose feeding was much lower than that of high fat feeding, which suggested that different calorie sources induced different extent effects on metabolism, even if they supplied same amount of calories.

Long-term excess energy intake is directly linked to the development of hyperlipidemia. Although the body weight of the fructose diet fed mice was no different to the control, the visceral fat weight and the number of liver fat droplets were much higher than in the control, which was consistent with a previous report in which the consumption of a fructose solution resulted in a significant increase in hepatic triglyceride accumulation in C57BL/6 mice [17]. However, the high fat-high fructose diet did not induce more lipid accumulation than the high fat alone diet. In a previous study, adult rats fed with high fructose-high fat for 2 weeks showed more deleterious effects on their lipid metabolism than those fed with high fat alone [18]; thus, fructose supplementation of a high fat diet might worsen the deleterious effects of short-term high fat feeding in C57BL/6 mice, which should be investigated further.

Saturated fat intake is associated with increased insulin resistance [19, 20]. Many studies have combined a high fat diet and fructose water to induce insulin resistance animal models [9]. Here, we compared the effects of high fat feeding and high fat-high fructose feeding on insulin resistance; however, the combined diet did not enhance the deleterious effects on insulin sensitivity obtained by high fat alone, which was consistent with the IGTT results in C57BL/6 mice obtained from a previous report [21]. Interestingly, according to the baseline glycemia difference in the IpITT experiment, fructose supplementation seems to increase the postprandial blood glucose concentration.

Glucose clearance rate is used commonly to evaluate insulin sensitivity in the short insulin tolerance test in humans [22]. The fall in glucose concentration within the initial 15 minutes after insulin injection is the most important [23]. In this study, we expanded the application of the glucose clearance rate from the insulin tolerance test to the glucose tolerance test. Accordingly, we observed a difference in the glucose disappearance rate between the IpGTT and IpITT. This might have been caused by (1) endogenous insulin (IpGTT) and exogenous insulin (IpITT) inducing different effects on glucose absorption; (2) endogenous insulin secreted by the pancreas was delayed in mice on an energy-dense diet, which should be investigated in future experiments.

Intake of high energy-dense foods could affect the basal metabolism. In this work, EE and RQ were affected by ingesting excess calories. Higher energy intake induced higher EE. The RQ is used to evaluate the utilization of carbohydrates, fats and proteins as energy suppliers *in vivo*. An RQ of 1.0 reflects carbohydrates oxidation and an RQ of <1.0 indicates oxidation of fat [24]. According to the RQ calculations, oxidation of fat was higher in mice fed with the high fat diet compared with the high fructose diet. Mice are nocturnal feeders; therefore, the RQ measured at night was higher than that in the day in the control mice and the high fructose mice. However, basal metabolism was impaired seriously in the high fat mice and high fat-high fructose mice. In these mice, lower RQ values were observed at night, suggesting that carbohydrates oxidation was affected by fat metabolism. In some studies, a high RQ (>1.0) has been observed under fructose treatment, caused by fructose-induced gluconeogenic and de novo lipid synthesis [25, 26], which was inconsistent with our results. This might reflect the difference in the duration of fructose treatment. Long-term fructose feeding induces lipid accumulation and fat oxidation increase.

Our work proved that excess energy intake was correlated with obesity, insulin resistance and metabolic disorders. However, we observed that the effect of changing the amount of energy intake on body weight gain was limited. The calorie source was also important for body weight and metabolism. We found that the energy efficiency of the 60% fat diet fed was significantly higher than that of the 20% fructose water fed. There are species differences in the deleterious effects of fructose feeding, and in this study, we did not observe that fructose supplementation worsened the detrimental effects of high-fat feeding on body weight or metabolism in C57BL/6 mice.
